# Standard Dyes for Total Protein Staining in Gel-Based Proteomic Analysis

**DOI:** 10.3390/ma3104784

**Published:** 2010-10-20

**Authors:** François Chevalier

**Affiliations:** Proteomic Laboratory, iRCM, CEA, Fontenay aux Roses, France; E-Mail: francois.chevalier@cea.fr; Tel.: +33-146-548-326; Fax: +33-146-549-138.

**Keywords:** protein staining, fluorescent dyes, two-dimensional electrophoresis, proteomics

## Abstract

Staining of two-dimensional gels is a primary concern in proteomic studies using two-dimensional gel electrophoresis with respect to the number of proteins analyzed, the accuracy of spot quantification and reproducibility. In this review article, the efficiency of the most widely used dyes was investigated. Visible dyes (Coomassie blue and silver nitrate), fluorescent dyes (Sypro Ruby, Deep Purple) and cyanine labeled methods were compared.

## 1. Introduction

**Figure 1:**
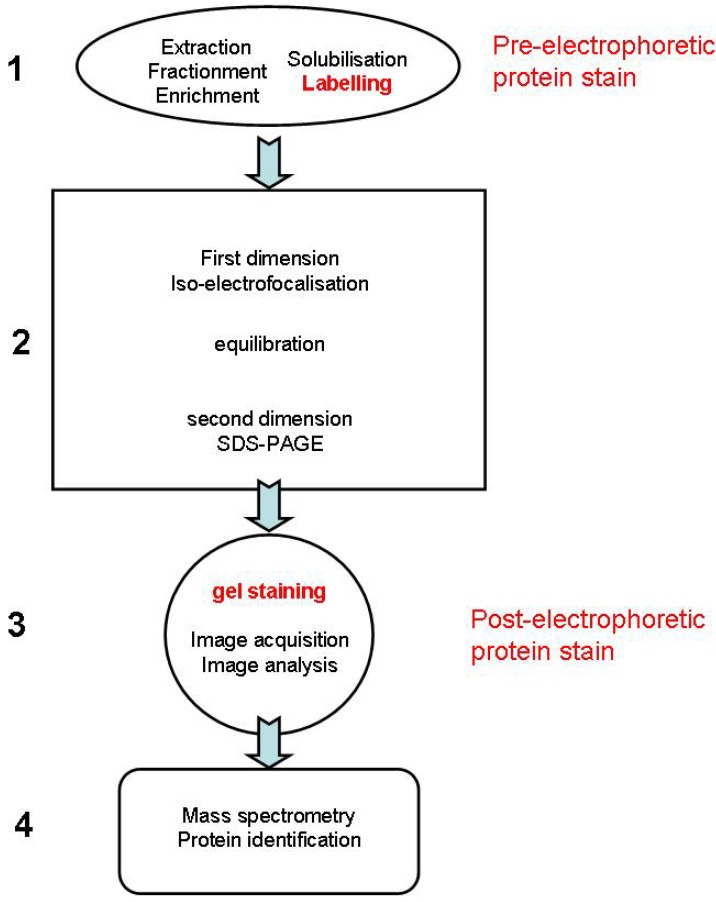
Pre-electrophoretic and post-electrophoretic protein staining during the different steps of 2DE.

Protein separation by two-dimensional electrophoresis (2DE) is largely used in proteomic approaches because of both high resolution and the availability of powerful image analysis software for gel comparison and compatibility with subsequent protein characterization by mass spectrometry [1]. For these various aspects, the selection of the protein staining procedure is of major importance [2]. Based on two independent biochemical characteristics of proteins, 2DE combines isoelectric focusing, which separates proteins according to their isoelectric point, and SDS-PAGE, which separates them further according to their molecular mass (**Figure 1, step 2**). The next typical steps of the flow of gel-based proteomics are spots visualization and evaluation (**Figure 1, step 3**), expression analysis, and finally protein identification by mass spectrometry (**Figure 1, step 4**). In order to take advantage of the high resolution capacity of 2DE, proteins have to be completely denatured, disaggregated, reduced and solubilized (**Figure 1, step 1**) to disrupt molecular interactions and to ensure that each spot represents an individual polypeptide. Proteins can be stained before the 2DE separation (pre-electrophoretic protein stain), or after 2DE separation (post-electrophoretic protein stain).

Classically, Coomassie blue was the most widely used non-covalent dye for post-electrophoretic protein staining [3]. However, it suffers from a low sensitivity in protein detection, including in the improved colloidal version [4]. In contrast, the other classical protein stain, silver nitrate, displays an excellent sensitivity but could interfere with protein analysis by mass spectrometry [5]. In the last decade, different fluorescent dyes have been introduced [6]. These encompass Sypro Ruby [7], and Ruthenium red-based dyes [8]. However, their present use remains relatively limited, probably due to their cost and/or technical difficulties. Recently, alternative molecules were proposed, including epicocconone, a natural fungal product [9].

Covalent protein labeling, called ‘‘difference gel electrophoresis’’ (DIGE) is nowadays an interesting alternative, which allowes multiplexing of samples and the use of an internal standard [10]. This pre-electrophoretic protein stain method leads to highly accurate qualitative and quantitative results, because gel-to-gel variations are eliminated.

The present review compares several non-covalent and covalent protein dyes for large-scale comparison of 2D gel electrophoresis patterns by image analysis in order to select the best method according to the biological question and sample type.

## 2. Post-Electrophoretic Protein Stains (see [11] for methods)

### 2.1. Coomassie Blue

**Figure 2:**
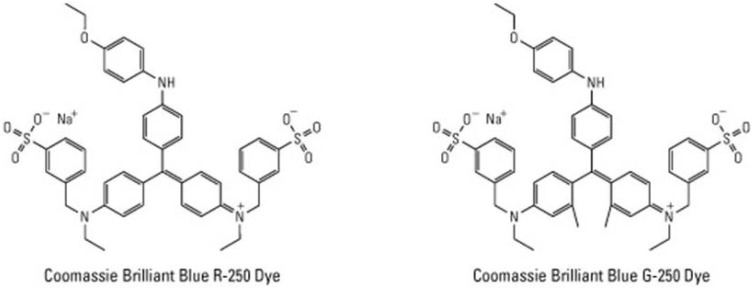
Chemical structures of Coomassie brilliant blue R-250 (left) and G-250 (right).

Two forms of Coomassie brilliant blue are available, R-250 and G-250 (**Figure 2**). R stands for reddish hue and G for greenish hue, the number 250 is an indicator number for dye strength. Typically, R-250 is used to stain SDS polyacrylamide gels and G-250 in the Bradford assay. The G-250 form allows an intensification of the stain with a low background in comparison with the R-250 form, thanks to an apparent conversion into a colloidal state in 12.5% TCA [4].

Coomassie brilliant blue R and G are known to interact differently with proteins. The exact mechanism of dye binding to the protein is not fully understood, but a significant effect can be expected from the dye’s net negative charge and its mostly nonpolar character. Interactions are chiefly with arginine rather than primary amino groups; the other basic (His, Lys) and aromatic residues (Try, Tyr, and Phe) give slight responses. The binding behavior is attributed to Van der Waals forces and hydrophobic interactions. Such non-covalent binding of the dye allowed an excellent compatibility with MALDI-TOF mass spectrometry [12].

**Figure 3:**
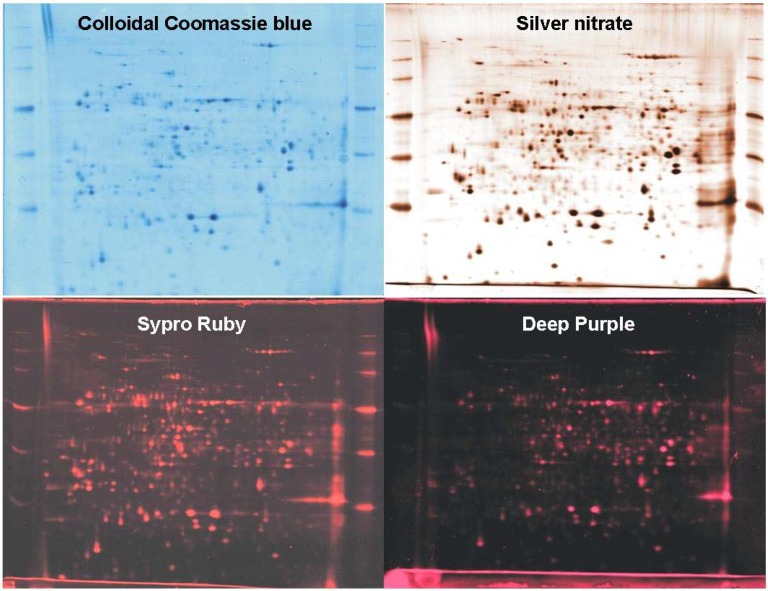
2D protein patterns of 100 µg of total protein extracts from *Arabidopsis*, focused on the pI 4–7 range, separated on gels covering the 15–150 kDa range and stained with colloidal Coomassie blue, silver nitrate, Sypro Ruby and Deep purple.

Colloidal Coomassie blue was observed as a judicious dye for large scale proteomic analysis [13]. It allowed a good identification of proteins regardless of their biochemical characteristics [14]. Nevertheless, on known proteins, the poor sensitivity of colloidal Coomassie blue (**Figure 3, top left**) contributed to a lack of information certainly due to a decrease of precision during spot picking. Moreover, an inconsistent mass compatibility was observed essentially for the weak concentration [15].

### 2.2. Silver Stain Methods

Two categories of silver staining were used to visualize proteins in gel: the acidic silver nitrate and the alkaline silver diamine procedure, which differed on binding specificity, sensitivity, cost and safety risk [7]. Numbers of modified protocols have been related since the first descriptions 25 years ago. Owing to the complex chemistry involved, many modifications were applied to decrease background and increase sensitivity [16]. More recently, efforts were oriented on the compatibility of silver stains with mass spectrometry [5]. Due to oxidative attack of silver ions on the proteins and to the use of various sensitizing pre-treatments of gels, irreversible modifications of amino acids have limited peptide mass fingerprint analysis or other mass spectrometry analysis. Most adaptations consisted of omitting cross–linking and sensitizing agents such as glutaraldehyde and formaldehyde [5], associated to a destaining method before enzymatic digestion.

The “Vorum silver staining protocol” of Mortz *et al*. [5] allowed a good sensitivity and a clear background (**Figure 3, top right**) [13]. Acidic proteins were less compatible to mass spectrometry analysis than neutral proteins. A large majority of the selected proteins stained with silver nitrate on the 100 µg gel were identified by peptide mass fingerprinting. This proportion decreased when proteins quantity was divided by two and by four. Sequence coverage of spots from gels left for 48 hours in water after staining and before excision decreased dramatically in comparison to experiments performed the same day as they were stained [17]. In our case, a two days computer analysis was performed before spots excision and analysis by mass spectrometry. This long storage period could explain the low number of matched peptides observed for the known proteins. Due to mediocre background homogeneity and to susceptibility to spot saturation [13], silver nitrate was not useful for quantitative and comparative proteomics analysis. Additionally, it was not possible to run an accurate image analysis prior to mass spectrometry since the spots had to be treated straight after staining. Gels stained with silver nitrate could be useful to obtain mass spectrometry results very quickly and with a low staining cost.

### 2.3. Sypro-Ruby

During the last decade, different fluorescent dyes were introduced and proved to combine high sensitivity and compatibility with mass spectrometry. These encompass both commercially available stains, such as the series of Sypros [18], and Ruthenium red-based dyes for which synthesis procedures were published [8]. However, their present use remains relatively limited, probably due to their cost and/or technical difficulties.

Sypro Ruby was described to combine sensitivity close to that of silver staining (**Figure 3, bottom left**) and the good properties of classical organic stains such as Coomassie blue [19]. Sypro Ruby is a luminescent ruthenium complex that interacts non-covalently with proteins thanks to a mechanism similar to the one of the colloidal Coomassie blue stain. As no irreversible modification of amino acids was operated during staining, satisfactory mass spectrometry compatibility was expected [18]. Sypro Ruby allowed stable sequence coverage regardless of spot intensity with a capacity of spots identification near the one of the colloidal Coomassie blue [15]. A constant identification of protein was shown independently of protein quantity. Additionally, Sypro Ruby was previously showed to have a broader linear dynamic range and a higher sensitivity than silver nitrate [13], suggesting a profitable use of this dye for large scale proteomic analysis. Nevertheless, the necessity of a fluorescent scanner added to the cost of the dye itself has limited the use of Sypro Ruby.

### 2.4. Epicocconone-Based Dyes

Deep Purple is a sensitive fluorescent-based stain [9] based on a natural compound extracted from the fungus *Epicoccum nigrum*. The fluorescent polyketide is able to bind to proteins and possibly to react on lysyl residues for fluorescence emission [20]. It was described to be more sensitive than Sypro Ruby and to be compatible with MALDI-TOF mass spectrometry [9]. The great sensitivity of Deep Purple was demonstrated previously, and is slightly less than Sypro Ruby, but with a weak susceptibility to background speckling (**Figure 3, bottom right**) [13]. A little less than half of the spots stained with Deep Purple were identified by mass spectrometry [15]. This was quite better than the spots stained with silver nitrate. Interestingly, as for Sypro Ruby and Silver Nitrate, Deep Purple seemed to be more compatible for mass spectrometry with proteins near neutral pH. On the other hand, no real influence of molecular weight was observed in the case of Deep Purple. Deep Purple showed better results than Sypro Ruby and the other staining techniques when the gels were loaded with a higher amount of proteins—in terms of numbers of matched peptides and identification of proteins. For this reason, Deep Purple could be recommended for 2DE gels staining followed by mass spectrometry analysis of abundant proteins [15].

## 3. Pre-Electrophoretic Protein Stains using Differential Gel Electrophoresis (DIGE)

Difference gel electrophoresis (DIGE) takes advantages of structurally similar cyanine-based dyes to label different pools of protein samples, which are then co-separated on a single 2DE gel [21].

**Figure 4:**
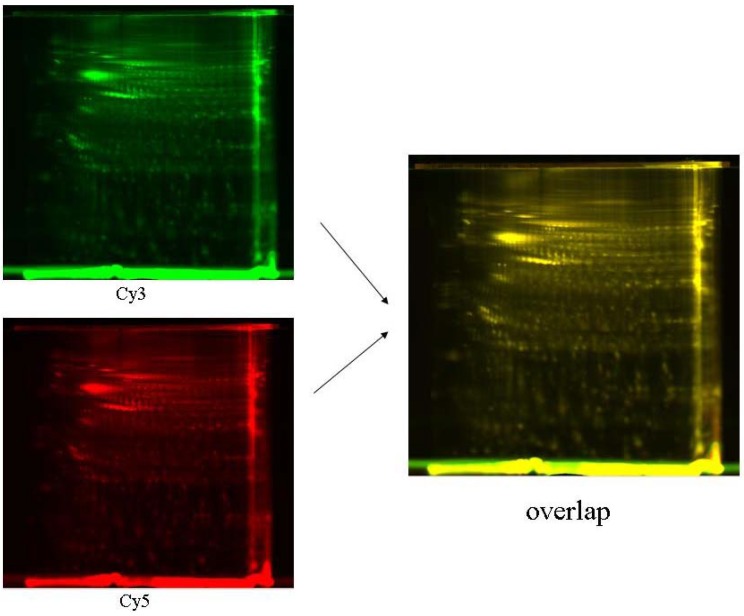
2D protein patterns of 5 µg of protein extracts from mouse brain stained by saturation labeling–DIGE, focused on pI 4–7 range, separated on gels covering the 15–150 kDa range.

The biggest advantage of DIGE over other 2DE-based technologies is that it enables the analysis of two or more protein samples simultaneously on a single 2DE (**Figure 4**). Since the same proteins present in two different samples were pre-labeled with two different dyes (*i.e.*, Cy3 and Cy5, respectively), they could be combined and separated on the same 2DE without the loss of the relative protein abundance in the original samples [10]. At the end of protein separation, the relative ratio of proteins in the two original samples could be readily obtained by comparing the fluorescence intensity of the same protein spots under different detection channels (e.g., Cy3 and Cy5) using a commercial fluorescence gel scanner. Because only one gel is used in DIGE, and the same proteins from two different protein samples co-migrate as single spots, there is no need for many replicates, making spot comparison and protein quantitation much more convenient and reliable. This makes DIGE potentially amendable for high-throughput proteomics applications [22].

DIGE has shown significant advantages over conventional 2DE in a number of applications. Up to three kinds of fluorescent cyanine dyes have been employed in DIGE, namely, Cy2, Cy3, and Cy5, which allows for simultaneous analysis of up to three different protein samples in a single gel. DIGE is a valuable method for high-throughput studies of protein expression profiles, providing opportunities to detect and quantify accurately “difficult” proteins, such as low-abundance proteins.

Typically, the labeling reaction is optimized such that only 1–5% of total lysines in a given protein are labeled. Alternatively, Shaw *et al*. have developed a new batch of DIGE Cy3 and Cy5 dyes, which label only free cysteines in a protein by “saturation” labeling [23]. This strategy offers greater sensitivity than the conventional DIGE method. The biggest drawback, however, is that it only labels proteins that contain free cysteines, meaning that a certain percentage of proteins in a proteome will not be labeled with this strategy, let alone downstream detection and characterization of these proteins.

**Table 1:** Comparison of analytical performances of standard dyes used for total protein staining in gel-based proteomic analysis. * cheap (+) or expensive (-).

	Colloidal Coomassie blue	Silver nitrate	Sypro Ruby	Deep Purple	DIGE
Type of stain	visible	visible	fluorescent	fluorescent	fluorescent
Post-electrophoretic	Yes	Yes	Yes	Yes	No
Pre-electrophoretic	No	No	No	No	Yes
Sensitivity	+	++	++	++	+++
Compatibility with MS	+++	+	+++	++	++
Reproducibility	++	+	++	++	+++
Cost*	+	+	-	-	-
Robustness for large scale analysis	++	+	+++	+++	+++

## 4. Conclusions

Several dyes are available to label proteins, either before or after electrophoresis. Some are quite expensive (Sypro ruby, Deep Purple, DIGE) while others are rather economical (colloidal Coomassie blue, silver nitrate) and affordable, as well as useful in protein identification by mass spectrometry (**Table 1**). For differential 2DE analysis it is important to obtain maximal information by combining non-covalent and covalent staining techniques. DIGE allows identification of faint spots from several samples, but in the future, the detection range of proteins needs to be considerably lowered to allow identification by mass spectrometry of such low abundance proteins.
